# Adrenal Cushing’s Syndrome in Pregnancy Complicated by Fetal Growth Restriction Following Retroperitoneoscopic Adrenalectomy

**DOI:** 10.7759/cureus.97934

**Published:** 2025-11-27

**Authors:** Masafumi Inoue, Rie Saito, Takayuki Sonoda, Masayuki Fujita, So Inamura, Yuko Kuroda, Saori Kaeriyama, Hiroshi Kawamura, Makoto Orisaka, Norio Harada, Yoshio Yoshida

**Affiliations:** 1 Obstetrics and Gynecology, University of Fukui School of Medical Sciences, Fukui, JPN; 2 Endocrinology and Metabolism, University of Fukui School of Medical Sciences, Fukui, JPN; 3 Urology, University of Fukui School of Medical Sciences, Fukui, JPN

**Keywords:** adrenal cushing’s syndrome, fetal growth restriction, hypercortisolism, pregnancy, retroperitoneoscopic adrenalectomy, superimposed preeclampsia

## Abstract

A 29-year-old Japanese pregnant woman, G5P3A1, conceived spontaneously and was referred to our hospital because of uncontrolled hypertension at 24 weeks of gestation. On admission, she presented with physical findings characteristic of Cushing's syndrome (CS), such as moon face, buffalo hump, and reddish-purple striae. Laboratory examination revealed hyperglycemia and hypercortisolism with suppressed adrenocorticotropic hormone levels. Imaging studies revealed a right adrenocortical adenoma, and the patient was clinically diagnosed with adrenal CS. At 28 weeks, she underwent retroperitoneoscopic adrenalectomy, which normalized maternal cortisol levels and improved metabolic abnormalities. Despite these improvements, she was diagnosed with fetal growth restriction accompanied by superimposed preeclampsia at approximately 33 weeks. The maternal serum soluble fms-like kinase 1 (sFlt-1)/placental growth factor (PlGF) ratio was markedly elevated. At 36 weeks, an emergency cesarean section was performed for fetal compromise, resulting in the delivery of a small-for-gestational-age infant. Histopathological examination of the placenta revealed ischemic changes consistent with placental insufficiency. Both the mother and infant were discharged in stable conditions. The present case shows that although adrenalectomy during pregnancy can correct endocrine abnormalities, it does not necessarily prevent subsequent fetal growth restriction.

## Introduction

Cushing’s syndrome (CS) is an endocrine disorder caused by chronic hypercortisolism. Because cortisol can disrupt ovulation, leading to menstrual irregularities and infertility [1,2], pregnancy in women with CS is exceedingly rare. Moreover, diagnosis during pregnancy is particularly challenging as many hallmark features of hypercortisolism - fatigue, weight gain, acne, and mood instability - are common in normal pregnancies.

Untreated CS during gestation is associated with substantially increased maternal and perinatal morbidity and mortality. Aggressive management during gestation, including cortisol synthesis inhibitors or surgical resection of pituitary adenomas or adrenal tumors, has been shown to improve maternal and fetal outcomes [3-5]. However, intensive treatment may not fully reduce the risks of fetal growth restriction and preterm delivery [5,6], and the underlying reason for this remains unclear.

Herein, we report a case of adrenal CS in a pregnant woman who underwent retroperitoneoscopic adrenalectomy at 28 weeks of gestation. Despite achieving biochemical remission of hypercortisolism after surgery, she developed fetal growth restriction and required preterm cesarean delivery due to fetal compromise.

This article was previously presented as a meeting abstract at (1) the 97th Annual Congress of the JES on June 7, 2024; (2) the 60th Annual Congress of JSPNM on July 15, 2024; and (3) the 47th Annual Meeting of JSGOS on November 24, 2024.

## Case presentation

A 29-year-old Japanese woman with a G5P3A1 conceived spontaneously. She had no medical history other than asthma and no particular familial history. She began receiving antenatal care at a nearby facility during the first trimester. She did not undergo screening tests for predicting the development of preeclampsia (PE), such as the first-trimester ultrasound at 11-14 weeks or pregnancy-associated plasma protein A assessment. Her casual blood glucose level was 87 mg/dL at 10^+6^ weeks of gestation. Initially, she was normotensive, but her blood pressure gradually increased to 144/100 mmHg at 18 weeks of gestation, and diagnosed as having chronic hypertension. Thereafter, her hypertension worsened, reaching 177/100 mmHg at 21 weeks of gestation, and she was diagnosed with superimposed PE. Around the same time, her body weight increased by 11.5 kg from the pre-pregnancy weight (from 58.5 kg to 70 kg), and generalized edema developed. As a result, she was admitted to the referring hospital and started taking antihypertensive treatment with oral methyldopa 750 mg/day, which lowered her blood pressure to a range of 130-150/80-100 mmHg, decreased her body weight to 66.5 kg, and improved the generalized edema. Although she was discharged from the hospital, her blood pressure increased again; thus, she was transferred to our institution, a tertiary referral perinatal medical center, at 24^+6^ weeks of gestation for subsequent perinatal management.

**Figure 1:**
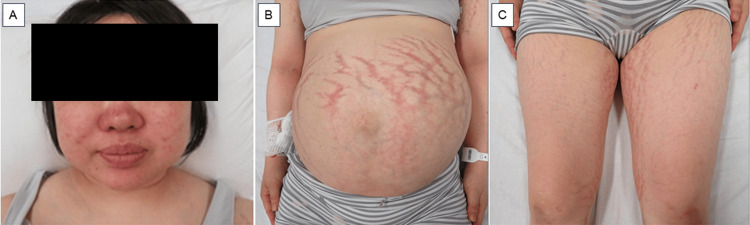
Macroscopic findings characteristic of Cushing’s syndrome (A) Moon face, (B) reddish-purple striae on abdomen, and (C) reddish-purple striae on thighs.

At her initial visit, her height and body weight were 153 cm and 66.2 kg, respectively. Her vital signs were as follows: body temperature 36.0℃, blood pressure 159/115 mmHg with the use of antihypertensive medication, and heart rate 80/min. She had an obvious full-moon face, acne vulgaris (Figure 1A), a buffalo hump, and reddish-purple striae on her abdomen and thighs (Figures 1B, 1C). She also had bilateral pitting edema in her lower legs and thin skin on the backs of her hands. No anemic palpebral conjunctiva, cervical lymphadenopathy, or thyroid enlargement was observed.

**Figure 2:**
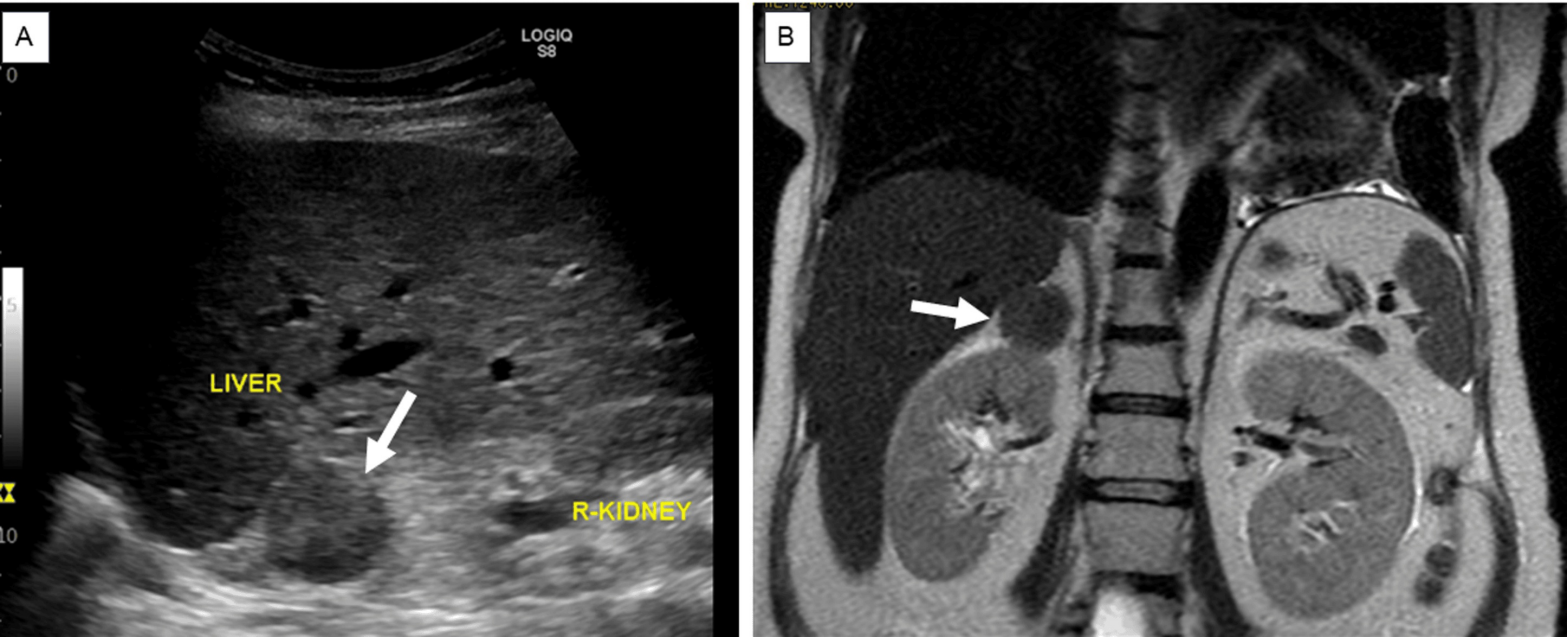
Radiological findings of the right adrenal tumor (white arrow) (A) Trans-abdominal ultrasonography image and (B) coronal section of the trunk on MRI.

**Figure 3:**
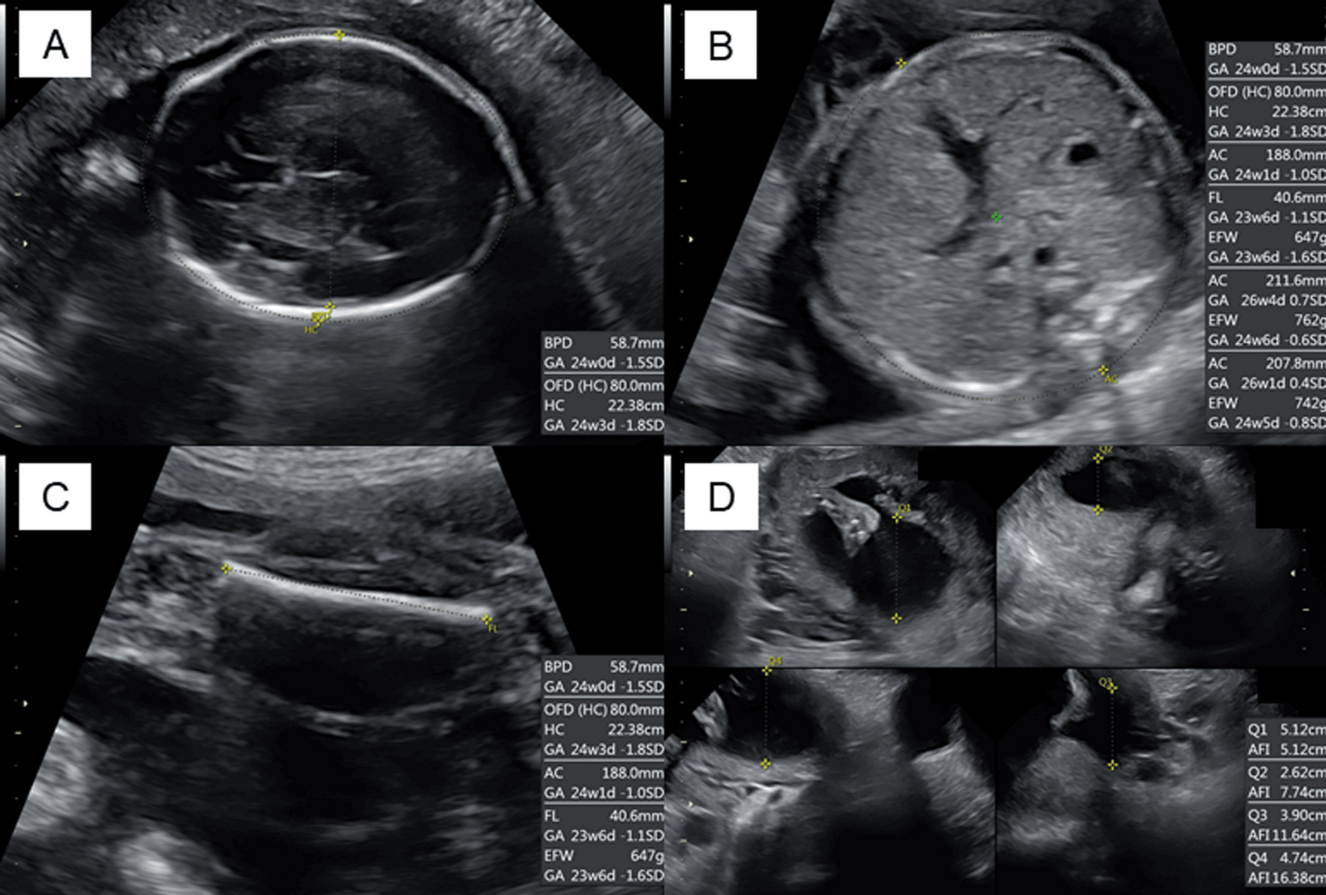
Obstetric ultrasonography (A) The plane used for biparietal diameter measurement, (B) the plane used for abdominal circumference measurement, (C) the plane used for femoral length measurement, and (D) the plane used for amniotic fluid index measurement.

An increased neutrophil count and decreased eosinophil count were observed, although the white blood cell count was within the normal range (Table 1). Biochemical analysis showed that the serum potassium level was decreased (2.3 mEq/L). The serum total protein, albumin, blood urea nitrogen, and cholinesterase levels were mildly decreased. Renal function, hepatic function, and lipid profiles were within normal limits, except for elevated triglyceride levels. A spot urine test indicated an elevated urine protein-to-creatinine ratio (0.436 g/gCr) (Table 2). Regarding diabetes-related tests, fasting plasma glucose (91 mg/dL), glycated hemoglobin (HbA1c) (5.4%), and glycated albumin (GA) (12.9%) were all within their normal ranges. The serum C-peptide level was elevated. A 75 g oral glucose tolerance test (OGTT) conducted at 25^+4^ weeks of gestation showed serum glucose levels of 191 mg/dL at one hour and 212 mg/dL at two hours (Table 2), indicating postprandial hyperglycemia. Endocrinological evaluation revealed elevated morning serum cortisol levels with loss of diurnal variation. This hypercortisolism is accompanied by suppressed plasma adrenocorticotropic hormone (ACTH) levels (Table 3). The 24-hour urinary free cortisol (UFC) level was markedly elevated (1,380 μg/day). In contrast, dehydroepiandrosterone sulfate (DHEA-S) levels decreased. Serum thyroid-stimulating hormone (TSH) was markedly decreased (0.091 IU/mL), accompanied by mild reductions in free T3 (1.65 pg/mL) and free T4 (0.65 ng/dL), which indicated central hypothyroidism. Abdominal ultrasonography revealed a nodule in the right adrenal gland with a maximum diameter of approximately 30 mm (28 × 27 × 25 mm) (Figure 2A). Abdominal magnetic resonance imaging (MRI) detected a 27-mm well-defined nodular lesion at the same location, which demonstrated a signal drop on opposed-phase images (Figure 2B). Obstetric ultrasonography revealed an estimated fetal body weight of 742 g (adequate for gestational age) (Figures 3A-3C), an amniotic fluid index of 16.4 cm (Figure 3D), and no major structural anomalies of the fetus. From the day of referral, oral nifedipine (40 mg/day) was initiated as antihypertensive therapy. Potassium chloride (KCl) was administered orally.

**Table 1:** Laboratory data of CBC, serum biochemistry, and endocrinology CBC: complete blood count, WBC: white blood cell count, Neut: neutrophil, Lymph: lymphocyte, Mono: monocyte, Eosino: eosinophil, RBC: red blood cell count, Hb: hemoglobin, Plt: platelet count, TP: total protein, Alb: albumin, T-Bil: total bilirubin, AST: aspartate aminotransferase, ALT: alanine aminotransferase, LDH: lactate dehydrogenase, ALP: alkaline phosphatase, γ-GTP: gamma-glutamyl transpeptidase, Na: sodium, K: potassium, Cl: chloride, Ca: calcium, P: phosphorus, BUN: blood urea nitrogen, UA: uric acid, Cr: creatinine, CRP: C-reactive protein, HDL-C: high-density lipoprotein cholesterol, LDL-C: low-density lipoprotein cholesterol, TG: triglyceride, FPG: fasting plasma glucose, HbA1c: hemoglobin A1c, GA: glycated albumin, C-peptide: connecting peptide, DHEA-S: dehydroepiandrosterone sulfate, TSH: thyroid-stimulating hormone, FT3: free triiodothyronine, FT4: free thyroxine

Parameter	Test value	Reference range
CBC		
WBC	8.1×10^9^/L	3.3-8.6 ×10^9^/L
Neut	83.5%	38.5-80.5%
Lymph	10.5%	16.5-49.5%
Mono	5.8%	2.0-10%
Eosino	0.1%	0.0-8.5%
RBC	3.17×10^12^/L	3.86-4.92 ×10^12^/L
Hb	11.5 g/dL	11.4-16.8 g/dL
Plt	190×10^9^/L	158-348 ×10^9^/L
Serum Biochemistry		
TP	5.7 g/dL	6.6-8.1 g/dL
Alb	3.3 g/dL	4.1-5.1 g/dL
T-Bil	1 mg/dL	0.4-1.5 mg/dL
AST	15 U/L	13-30 U/L
ALT	27 U/L	7-23 U/L
LDH	326 U/L	124-222 U/L
ALP	55 U/L	38-113 U/L
γ-GTP	29 U/L	9-32 U/L
Na	146 mEq/L	138-145 mEq/L
K	2.3 mEq/L	3.6-4.8 mEq/L
Cl	107 mEq/L	101-108 mEq/L
Ca	8.5 mg/dL	8.8-10.1 mg/dL
P	2.1 mg/dL	2.7-4.6 mg/dL
BUN	6 mg/dL	8-20 mg/dL
UA	3.4 mg/dL	2.6-5.5 mg/dL
Cr	0.45 mg/dL	0.46-0.79 mg/dL
CRP	0.1 mg/dL	0-0.14 mg/dL
HDL-C	66 mg/dL	48-103 mg/dL
LDL-C	134 mg/dL	65-163 mg/dL
TG	211 mg/dL	30-117 mg/dL
FPG	91 mg/dL	73-109 mg/dL
HbA1c	5.4%	4.9-6.0%
GA	12.9%	12.3-16.5%
C-peptide	3.7 ng/mL	0.6-1.8 ng/mL
Endocrinology		
Adrenaline	<0.01 ng/mL	<0.17 ng/mL
Noradrenaline	0.09 ng/mL	0.15-0.57 ng/mL
Dopamine	<0.02 ng/mL	<0.03 ng/mL
Cortisol	24.7 μg/dL	3.7-19.4 μg/dL
Aldosterone	<4.0 pg/mL	4.0-82.1 pg/mL
Renin activity	0.7 ng/mL/hr	0.2-3.9 ng/mL/hr
DHEA-S	43 μg/dL	92-399 μg/dL
TSH	0.091 IU/mL	0.350-4.940 IU/mL
FT3	1.65 pg/mL	1.68-3.67 pg/mL
FT4	0.65 ng/dL	0.70-1.48 ng/dL

**Table 2:** Laboratory data of 75-g OGTT and urinalysis OGTT: oral glucose tolerance test, PG: plasma glucose, IRI: immunoreactive insulin, U-Cr: urinary creatinine, U-TP: urinary total protein

Parameter	Test value
75-g OGTT	
PG	
0 min	91 mg/dL
30 min	153 mg/dL
60 min	191 mg/dL
90 min	204 mg/dL
120 min	225 mg/dL
IRI	
0 min	10.8 μU/mL
30 min	29.3 μU/mL
60 min	48.7 μU/mL
90 min	64.6 μU/mL
120 min	91.2 μU/mL
Urinalysis	
U-Cr	39 mg/dL
U-TP	17 mg/dL
U-TP/Cr	0.436 g/gCr

**Table 3:** Laboratory data of ACTH/F diurnal rhythm ACTH: adrenocorticotropic hormone, F: cortisol

Parameter	Test value	Reference range
ACTH/F diurnal rhythm		
ACTH		
6:00 AM	2.1 pg/mL	7.2-63.3 pg/mL
4:00 PM	2.0 pg/mL	7.2-63.3 pg/mL
11:00 PM	2.3 pg/mL	7.2-63.3 pg/mL
F		
6:00 AM	24.7 μg/dL	3.7-19.4 μg/dL
4:00 PM	25 μg/dL	3.7-19.4 μg/dL
11:00 PM	25.8 μg/dL	3.7-19.4 μg/dL

**Figure 4:**
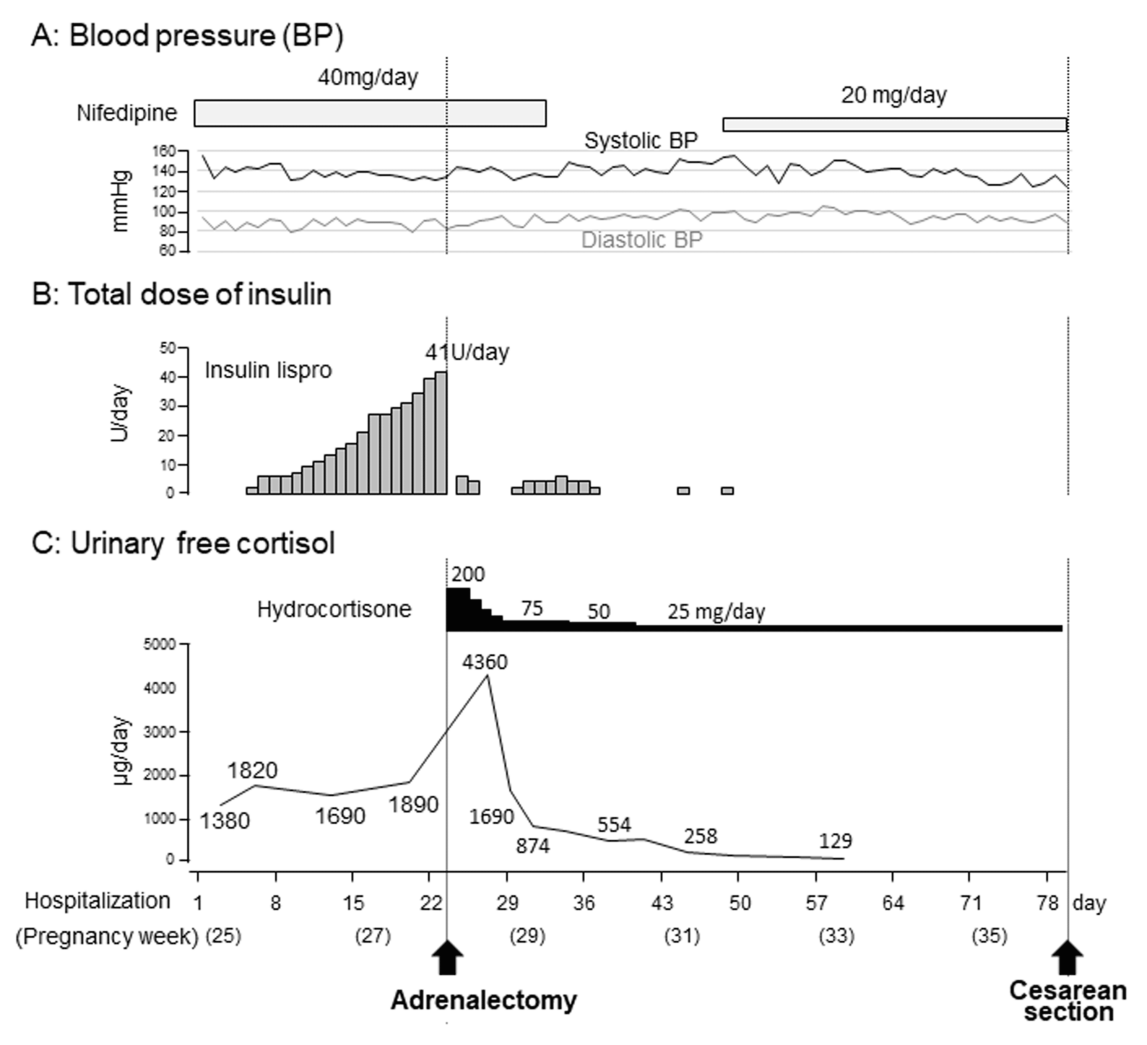
Clinical course between hospitalization and cesarean delivery (A) Blood pressure trend, (B) total dose of insulin, and (C) urinary free cortisol trend.

Physical examination revealed typical signs of CS, such as a moon face, buffalo hump, and reddish-purple striae. In addition, laboratory findings showed elevated UFC, increased nocturnal serum cortisol levels (>5.0 μg/dL), and suppressed ACTH levels (<5.0 pg/mL). On the basis of these findings, the patient was diagnosed with ACTH-independent CS. Furthermore, imaging studies identified a right adrenal mass, leading to a final diagnosis of CS caused by a right adrenal tumor. Both central hypothyroidism and impaired glucose tolerance were considered secondary complications, primarily caused by hypercortisolemia due to CS. The serum potassium level was maintained at approximately 3.0 mEq/L after the administration of oral KCl. An increase in the nifedipine dose from 20 mg/day to 40 mg/day stabilized the blood pressure at approximately 140/90 mmHg (Figure 4A). Intensive insulin therapy with insulin lispro was initiated on hospital day 4 (Figure 4B), and the insulin dosage was gradually increased for postprandial hyperglycemia. The maximum insulin dose was 41 units/day on day 23 of hospitalization. Throughout this period, the UFC levels remained persistently elevated (Figure 4C).

**Figure 5:**
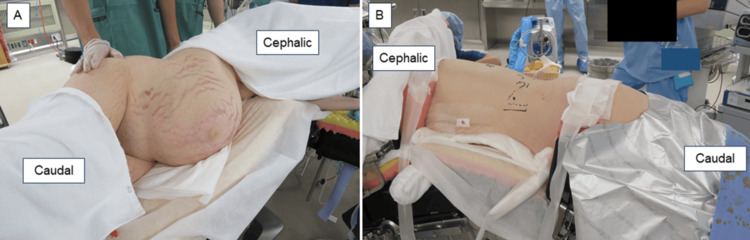
Photograph showing the patient in the left lateral decubitus position after general anethesia (A) Abdominal area and (B) dorsal area.

**Figure 6:**
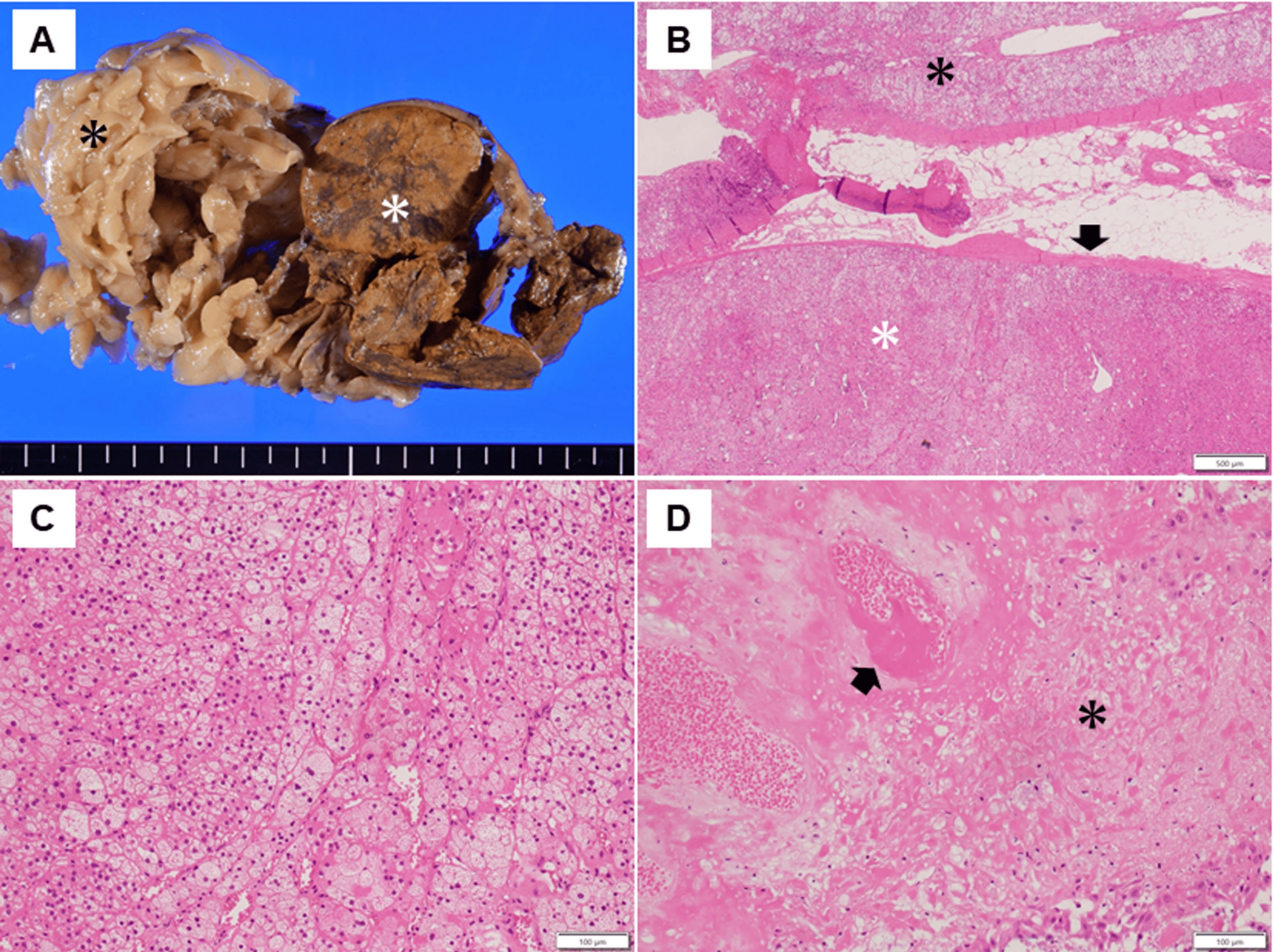
Histopathological findings of the right adrenal gland (A, B, C) and placenta (D) (A) Macroscopic view of the right adrenal gland showing the normal adrenal tissue (black asterisk) and the adrenal tumor (white asterisk). (B, C) Microscopic findings of the right adrenal gland and tumor (H&E staining). (B) Normal adrenal gland (black asterisk) and adrenal tumor (white asterisk) separated by a thin fibrous capsule (black arrow). (C) Tumor cells with abundant eosinophilic to clear cytoplasm arranged in a trabecular to microacinar growth pattern. (D) Microscopic findings of the placenta (H&E staining) showing fibrin deposition within villous vessels (black arrow) and chorionic villi with loss of nuclear detail and crowding (black asterisk).

A clinical team of obstetricians, urologists, and endocrinologists discussed the treatment plans for CS and perinatal management. Pharmacological treatment had two problems: radicality and risk of fetal adrenal insufficiency due to placental passage of medication; therefore, we decided to perform adrenalectomy during pregnancy. At 28^+3^ weeks of gestation, a retroperitoneoscopic adrenalectomy was performed by urologists. After the induction of general anesthesia, the patient lay on the bed in a complete left lateral position (Figures 5A, 5B). Consequently, the endoscope and instrument ports were placed in the same configuration as those used in the conventional retroperitoneal approach for nonpregnant patients. Port placements were planned guided by abdominal ultrasonography to identify the uterine position, and the assistant port was positioned at a location that minimized potential interference with the uterus. The surgery was completed without complications. The operative time was 83 minutes, and bleeding was minimal. Histopathological examination indicated that the tumor was an adrenocortical adenoma (Figures 6A-6C).

**Figure 7:**
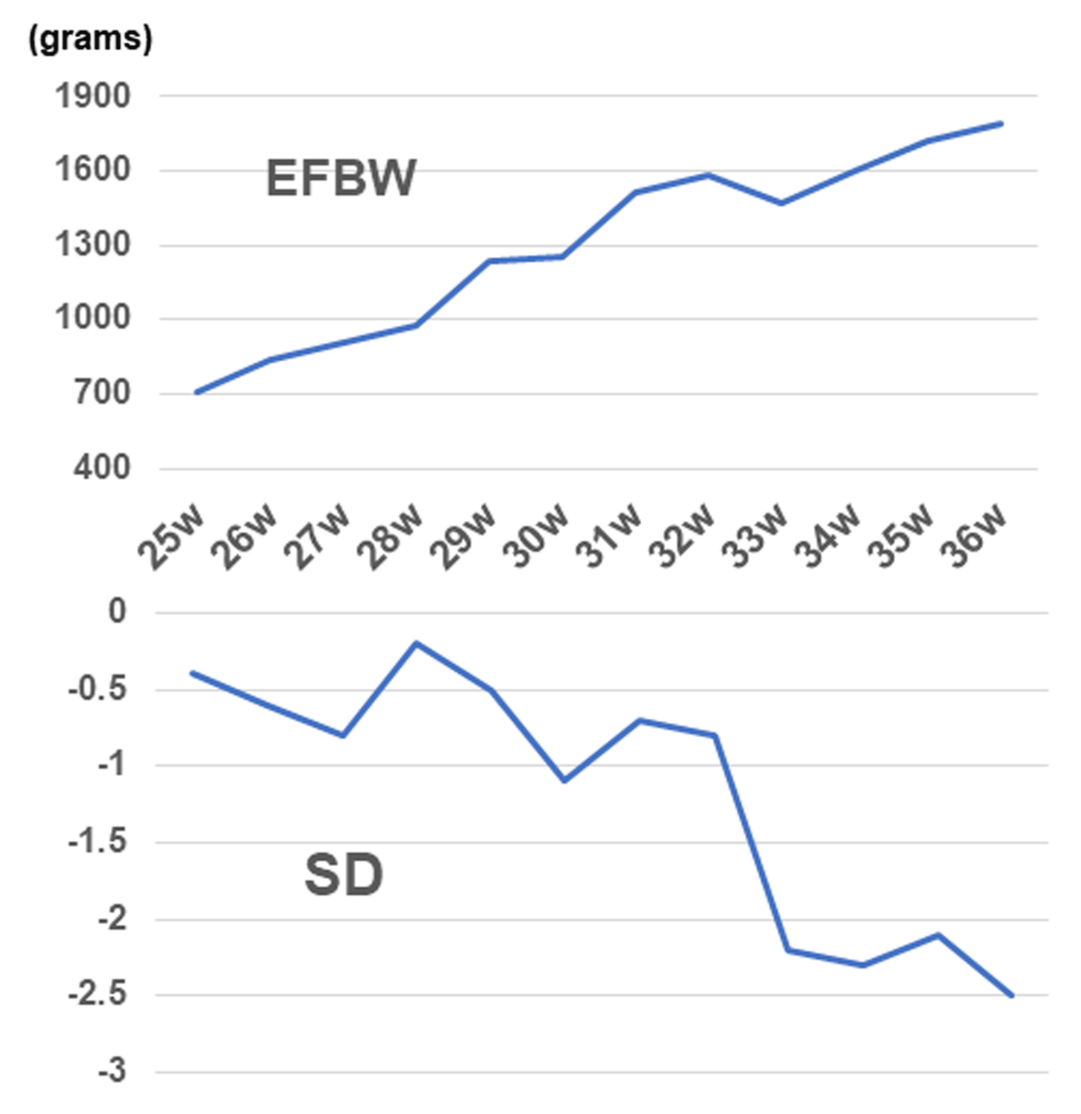
Trends in estimated fetal body weight (EFBW) and standard deviation (SD)

After surgery, the maternal glucose tolerance rapidly improved, and intensive insulin therapy with insulin lispro became unnecessary (Figure 4B). To avoid postoperative adrenal insufficiency, replacement therapy with hydrocortisone was initiated at 200 mg/day immediately after surgery, and the dosage was gradually tapered to 25 mg/day before delivery (Figure 4C). Maternal thyroid function normalized two weeks after surgery. At 29 weeks of gestation, oral nifedipine (40 mg/day) was stopped and blood pressure was monitored; however, high blood pressure was sustained. Therefore, oral nifedipine was resumed at 20 mg/day at 31 weeks of gestation. At approximately 33 weeks of gestation, the fetus exhibited slow growth, leading to a diagnosis of fetal growth restriction. The levels of serum soluble fms-like kinase 1 (sFlt-1)/placental growth factor (PlGF) were 173 (7990/46.1) at 33^+0^, 299 (11600/38.9) at 34^+1^, and 316 (15200/48.1) at 35^+5^ weeks. Trends in the estimated fetal body weight and standard deviation are shown in Figure 7. At 36^+1^ weeks of gestation, cardiotocography revealed severely prolonged deceleration regardless of the absence of uterine contraction, and an emergency cesarean section was performed. A male infant weighing 1,726 g and 41 cm in height, diagnosed as small for gestational age, was born with Apgar scores of 8 at one minute and 9 at five minutes. The umbilical arterial pH was 7.36. The size and weight of the placenta were 14.7 × 12.8 × 3.0 cm and 315 g, respectively, and histopathological examination revealed findings consistent with ischemic infarction (Figure 6D). Antihypertensive drugs administered to the mother were discontinued on day 8. The mother and neonate were discharged on POD 20. The child achieved normal development at the age of two years.

## Discussion

This case illustrates adrenal CS in pregnancy, complicated by the subsequent development of fetal growth restriction, despite retroperitoneoscopic adrenalectomy at 28 weeks of gestation. Notably, a markedly increased maternal serum sFlt-1/PlGF ratio was detected at the time of diagnosis of fetal growth restriction. To the best of our knowledge, this is the first case in which angiogenic markers were evaluated in a pregnant woman with adrenal CS.

The coexistence of CS and pregnancy is extremely rare [4]. The primary reason for this rarity is infertility, often caused by the hypercortisolism characteristic of CS. Specifically, hypercortisolism suppresses the hypothalamic-pituitary-gonadal axis, leading to impaired follicular development and anovulation by disrupting the secretion of gonadotropin-releasing hormone (GnRH) [1,7]. Pregnancy poses significant challenges in patients with ACTH-dependent CS, in whom excessive ACTH production is accompanied by androgen overproduction. As a result, adrenal etiologies of CS are more common than pituitary-dependent etiologies during pregnancy [3]. Several factors make it difficult to diagnose CS during pregnancy. First, the characteristic physical findings of CS closely mimic physiological changes in normal pregnancy. For example, weight gain, abdominal striae, and edema are common symptoms of both conditions. Therefore, this overlap can cause delayed diagnosis or misdiagnosis of CS during pregnancy [3]. It has been reported that 21.5% of pregnant women with CS are diagnosed only after delivery [3]. Second, physiological hormonal changes during pregnancy complicate the diagnostic process. During gestation, the placenta produces corticotropin-releasing hormone (CRH) and ACTH [8]. Additionally, elevated estrogen levels increase the synthesis of corticosteroid-binding globulin, resulting in a state of physiological hypercortisolism in pregnant women [9,10]. Consequently, the dexamethasone suppression test, which is key to the diagnosis of CS, is often unreliable in pregnant women because of the high incidence of false-positive results [4].

Despite these diagnostic hurdles, certain findings are highly valuable in identifying CS during pregnancy. First, careful examination of physical signs specific to CS, such as skin thinning and the presence of wide, reddish-purple striae, is crucial. Second, the evaluation of diurnal cortisol rhythms was informative. While this rhythm is preserved in normal pregnancy, it is characteristically absent in CS. Therefore, measuring late-night serum cortisol levels is useful for differentiating between these two states [11]. Third, a 24-hour UFC level exceeding three times the upper limit of normal for non-pregnant individuals is strongly suggestive of CS [4,7,9]. In the present case, these key features were decisive for the diagnosis. We found wide, reddish-purple striae, a loss of diurnal cortisol rhythm, and a markedly elevated 24-hour UFC level. Based on these findings, we definitively diagnosed the patient with CS complicating pregnancy.

According to a systematic review of 263 pregnancies complicated by CS, untreated pregnant women were significantly more likely to develop PE than those treated beforehand (26.5% vs. 2.3%) [3]. PE is characterized by defective placentation and impaired spiral artery remodeling, leading to placental ischemia during early pregnancy. Placental ischemia produces sFlt-1, a splice variant of Flt-1 that binds to vascular endothelial growth factor and PlGF and serves as a biochemical marker of endothelial dysfunction that inhibits angiogenesis [12]. Systemic endothelial dysfunction leads to maternal hypertension, proteinuria, and damage to other organs, including the placenta. In this case, placental histopathology indicated ischemic changes without retroplacental hematoma. In addition, a marked elevation of the sFlt-1/PlGF ratio - resulting from both increased sFlt-1 and decreased PlGF - was detected, supporting the presence of placental ischemia due to impaired placentation in early pregnancy.

In this case, several factors may have contributed to the placental ischemia. First, poor control of maternal hyperglycemia or hypertension may have played a role. As hyperglycemia is known to induce oxidative stress [13], it is possible that hyperglycemia in early pregnancy causes placental ischemia indirectly via oxidative stress. Recent studies suggest that hypertension in early pregnancy may contribute to impaired placentation, thereby increasing the risk of subsequent superimposed PE [14,15]. Therefore, chronic hypertension associated with CS may also be related to placental ischemia, although the maternal outpatient blood pressure was within the normal range during early pregnancy in the present case. Second, chronic hypercortisolemia can directly contribute to abnormal placentation. Previous animal experiments have shown that elevated maternal serum cortisol levels enhance uterine arterial contractions [16], which may induce placental ischemia. Furthermore, chronic hypercortisolism may exceed the protective capacity of 11β-hydroxysteroid dehydrogenase type 2 (11β-HSD2), which shields the fetus from excessive cortisol, thereby directly affecting the fetus [17]. Based on these findings, it is presumed that irreversible placental damage had already occurred at the time of the surgical resection in this case. Preconceptional or at least early diagnosis and treatment of CS are crucial for preventing fetal growth restriction associated with superimposed PE after surgery.

The second trimester is generally considered the optimal period for adrenalectomy in pregnant patients with adrenal CS [18]; however, successful procedures have been reported even during the third trimester [6,19]. Endoscopic adrenalectomy is favored over open approaches owing to its reduced morbidity, although direct comparisons between the transperitoneal and retroperitoneal approaches in pregnancy are lacking. In non-pregnant patients, both approaches yield similar operative times, blood loss, and hospital stays [20]. In this case, the retroperitoneal approach was used. This technique offers several advantages during pregnancy as follows: it allows surgery in the lateral position, minimizes inferior vena cava compression by the gravid uterus, avoids entry into the peritoneal cavity, thereby preventing interference from the enlarged uterus, and reduces the risk of intra-abdominal inflammatory spread to the uterus and adjacent organs. Based on our experience and considering the potential advantages of the retroperitoneoscopic approach, we propose that retroperitoneoscopic adrenalectomy should be considered even in the early third trimester, as it may safely prolong gestation and reduce the need for preterm delivery.

## Conclusions

This case highlights the challenges of managing adrenal CS during pregnancy. Uncontrolled CS may impair placental development during early pregnancy; therefore, preconceptional or at least early recognition and appropriate management are crucial to minimize the risk of subsequent fetal growth restriction. Further research is needed to clarify the pathophysiological relationship between hypercortisolism and impaired placentation in early pregnancy and to refine strategies for managing this rare but high-risk condition.
